# Privacy-Centric AI and IoT Solutions for Smart Rural Farm Monitoring and Control

**DOI:** 10.3390/s24134157

**Published:** 2024-06-26

**Authors:** Mosiur Rahaman, Chun-Yuan Lin, Princy Pappachan, Brij B. Gupta, Ching-Hsien Hsu

**Affiliations:** 1 International Center for AI and Cyber Security Research and Innovations (CCRI), Asia University, Taichung 413, Taiwan; mosiurahaman@gmail.com; 2 Department of Computer Science and Information Engineering, Asia University, Taichung 413, Taiwan; cyulin@asia.edu.tw; 3 Center for the Development of Language Teaching and Research, Asia University, Taichung 413, Taiwan; princypappachan.p@gmail.com; 4 Symbiosis Centre for Information Technology (SCIT), Symbiosis International University, Pune 412115, India; 5 Center for Interdisciplinary Research, University of Petroleum and Energy Studies (UPES), Dehradun 248007, India; 6 Guangdong-Hong Kong-Macao Joint Laboratory for Intelligent Micro-Nano Optoelectronic Technology, School of Mathematics and Big Data, Foshan University, Foshan 528000, China; 7 Department of Medical Research, China Medical University Hospital, China Medical University, Taichung 327, Taiwan

**Keywords:** smart farming, artificial intelligence, sensors technology, security

## Abstract

The integration of artificial intelligence (AI) and the Internet of Things (IoT) in agriculture has significantly transformed rural farming. However, the adoption of these technologies has also introduced privacy and security concerns, particularly unauthorized breaches and cyber-attacks on data collected from IoT devices and sensitive information. The present study addresses these concerns by developing a comprehensive framework that provides practical, privacy-centric AI and IoT solutions for monitoring smart rural farms. This is performed by designing a framework that includes a three-phase protocol that secures data exchange between the User, the IoT Sensor Layer, and the Central Server. In the proposed protocol, the Central Server is responsible for establishing a secure communication channel by verifying the legitimacy of the IoT Sensor devices and the User and securing the data using rigorous cryptographic techniques. The proposed protocol is also validated using the Automated Validation of Internet Security Protocols and Applications (AVISPA) tool. The formal security analysis confirms the robustness of the protocol and its suitability for real-time applications in AI and IoT-enabled smart rural farms, demonstrating resistance against various attacks and enhanced performance metrics, including a computation time of 0.04 s for 11 messages and a detailed search where 119 nodes were visited at a depth of 12 plies in a mere search time of 0.28 s.

## 1. Introduction

Rural farming practices have been a significant driving force in the evolution of agriculture, progressing from the use of simple tools to the advanced deployment of automated equipment which has boosted productivity and enhanced efficiency. The era of traditional farming, rooted in manual labor, animal power, and rudimentary farming techniques sensitive to environmental conditions, has faced persistent challenges related to efficiency, scalability, and sustainability. However, in recent years, rural farming has steadily begun to include technological advancements and innovations to overcome the inherent limitations of traditional farming practices [1]. This continuous integration of technology reached a pivotal transformation with the advent of smart farming, referred to as Agriculture 4.0, a modern approach that uses the Internet of Things (IoT), artificial intelligence (AI), cloud computing, and other advanced hardware and software to revolutionize agricultural practices [2].

Smart farming employs various sensors and gadgets to continuously gather and communicate vast amounts of data about crop health, weather patterns, soil conditions, and machine learning algorithms that predict crop diseases and optimize harvesting schedules [3]. These sensors connected to the IoT are the driving force behind smart farms, as they collect data in real time and process it locally or on a cloud server using advanced AI algorithms to offer farmers access to previously unimaginable insights and decision-making tools [4].

Studies on smart farming have focused on hardware components like sensors, unmanned aerial vehicles (UAV), global positioning systems (GPSs), and automated machinery and software components that involve data analysis platforms, decision support systems, and mobile applications that process and interpret collected data to aid in decision-making. Innovations in this field involve increasing sensor accuracy, refining the software’s data processing capabilities, and improving UAVs’ operational efficiency.

However, despite these technological advancements and increased academic and commercial research on smart farming, there is a significant gap in the research on privacy-centric solutions, necessary for rural farm monitoring and control. This is particularly important as deploying AI and IoT in rural settings introduces complex challenges in data security and privacy management due to limited cybersecurity infrastructures. Additionally, with more advanced and linked technologies being used, processing and storing agricultural data has become more and more dependent on cloud-based infrastructure due to the growth in data collection, which raises privacy and security concerns about sensitive agricultural data. This definition of “data” includes farmers’ financial and personal information, including meteorological patterns and statistics on agricultural productivity, which could be compromised if insufficient security precautions are implemented [5]. Furthermore, considering most farmers are unfamiliar with digital security, they are ill-equipped to tackle the complexities of safeguarding their digital information [6].

Conventionally, in smart farming, IoT sensors, cloud servers, and human experts are the three primary nodes that must be managed effectively. IoT sensors positioned systematically throughout the farm serve as the primary data collection points, gathering accurate details about crops and the surrounding environment [7]. On the other hand, cloud sensors act as the primary hub for data processing and storage, utilizing AI algorithms to analyze the gathered data and produce informative results. Within this system, the human expert, typically a farmer or farm manager, collects information, makes defendable judgments, and sometimes manually controls or modifies IoT devices in response to ideas given [8].

Our research thus focuses on developing a secure and privacy-centric framework for smart rural farm monitoring and control by proposing a protocol that ensures secure data exchange across different layers in the smart farm AI–IoT architecture. This protocol is designed to encrypt data, establish a secure communication channel, verify the legitimacy of all nodes, and restrict access exclusively to authorized entities. Implementing such a layer of protection is essential for averting catastrophic consequences and protecting farm operational data from unwanted access and data breaches [9]. To test the robustness and efficiency of the proposed protocol, we use the Automated Validation of Internet Security Protocols and Applications (AVISPA) tool, an accepted standard for verifying and validating security protocols [10,11]. Our objective is thus to develop a framework that enables the integration of AI and IoT technologies in rural agriculture while ensuring the security and privacy of all participants and to add to the existing literature on smart farming by offering insights and recommendations that can enhance the security of smart rural farms.

The rest of the article is organized into seven main sections: Section 2 discusses related works in smart farming and advanced technological solutions in smart farming. This is followed by a discussion of the integration of AI and IoT in smart farming in Section 3 and a discussion of privacy concerns in AI and IoT in smart farming in Section 4. Section 5 introduces our protocol, which involves three entities: the User, the IoT Sensor Layer, and the Central Server. Section 6 addresses the formal verification of the proposed protocol using the AVISPA tool, presents the results of the simulation, and compares these results with other existing security protocols. Finally, Section 7 summarizes and presents the conclusions outlining the future scope of AI–IoT research in smart rural farm monitoring and control.

## 2. Related Work

Numerous studies have been conducted to develop systems that employ AI and IoT for environmental monitoring, crucial for agriculture decision-making. These technologies also form part of precision agriculture, ensuring that resources are used efficiently, minimizing waste, and maximizing output. These systems typically use sensors to gather data on environmental parameters like temperature, humidity, rainfall, and barometric pressure [12]. Several studies have also focused on developing smart irrigation systems to optimize water usage, prevent resource wastage, and adjust to the crop’s specific hydration requirements. For instance, soil moisture sensors help determine the most effective watering schedules to prevent over- or under-watering. Similarly, pH sensors that monitor soil acidity can influence nutrient availability and absorption, while temperature and humidity sensors help predict frost events or identify conditions conducive to fungal infections. Additional research has looked into decision support systems (DSS) that use AI algorithms to provide insight into pest management issues and crop rotation recommendations for sustainability [2].

In all these studies, the collected data from sensors are subsequently sent to cloud servers for storage and analysis. This employment of cloud computing facilitates a faster, easier, and more maintainable monitoring process. The application of this technology also allows seamless collaboration among stakeholders and provides farmers with a holistic view of their operations. These numerous advantages of utilizing cloud computing via data collection and management with AI and IoT integration allow farmers to access advanced technologies and data analysis tools that improve productivity, efficiency, and sustainability. However, these advantages are accompanied by challenges such as data security, reliable internet connectivity, and the need for technical expertise.

Key security concerns include data breaches and distributed denial of service (DDoS), where unauthorized access to sensitive farm data can disrupt farming operations. Other dangers that can compromise data security involve data loss due to technical failures, accidental deletions, inadequate backup practices, and insider threats from workers or third-party suppliers with access to cloud systems. Additionally, data sharing and third-party access can result in farm data being accessed and shared without explicit consent, leading to potential misuse or unauthorized disclosures [13]. Moreover, there are worries over transparency and the possible misuse of personal data because the AI algorithms that process and evaluate this data frequently function as “black boxes” [14].

To address these concerns, various methods of protecting privacy have been created and applied in comparable situations. Three notable methods are federated learning, which trains AI models across multiple decentralized devices or servers holding local data samples without exchanging them; data anonymization, which removes personally identifiable information from data sets; and encryption, which secures data both in transit and at rest [15]. Another potentially effective method is secure multi-party computation, which allows individuals to collaboratively compute a function based on their inputs while preserving the privacy of those inputs [16]. However, many existing solutions are too resource-intensive or excessively complicated to use in rural locations where resources might be scarce [17]. Furthermore, the lack of technical expertise hinders the effective use of platforms that integrate these various AI and IoT innovations, which calls for reliable, user-friendly, and efficient confidential solutions adaptable to rural farming requirements [17,18].

Several studies have addressed these concerns in smart farming by proposing different security mechanisms, such as privacy-oriented blockchain-based solutions in green IoT-based agriculture, authentication and access control, symmetric data encryption between agricultural sensors, intrusion detection systems, and physical countermeasures. Early studies involved smartcard and password-based protocols to ensure the security of wireless sensor networks, which proved ineffective against evolving threats such as insider and impersonation attacks.

Some traditional solutions also depended on a trusted third party for device identity management, introducing risks such as single points of failure and security threats from third parties [19]. Subsequent research introduced protocols that addressed mutual authentication and the absence of password change phases. However, these protocols were also vulnerable to impersonation attacks and weaknesses such as session key leakage and offline password guessing attacks. So, with the further advancement of technologies and the IoT, blockchain-based authentication and key agreement schemes were developed, which unfortunately suffered from high costs. Recent research then prioritized using elliptic curve cryptography (ECC) and other advanced cryptographic techniques to create security protocols to address the various vulnerabilities.

The use of the AVISPA tool also emerged as a useful tool in testing the robustness of security protocols with the integration of the Dolev–Yao (DY) threat model in AVISPA during the simulation of security protocols. Since the DY model assumes that an adversary can intercept, modify, and fabricate messages, AVISPA can effectively test protocols under worst-case scenarios.

## 3. Integration of AI and IoT in Smart Farming

By 2050, the human population is estimated to be 9.4 to 10.1 billion, which necessitates an increase in food production, particularly planting and livestock production. According to the Food and Agricultural Organization (FAO), global food systems will be unable to keep up with this population growth unless substantial changes have been made towards agricultural productivity. However, environmental changes caused by human activities have endangered the agrarian sector, while urbanization has decreased labor availability in traditional food production areas. Considering how food production must increase by one-third over the current level to meet the growing population demand, smart farming has emerged as an answer to these challenges by employing diverse technologies across various levels and stages of agriculture production. Another notable factor is how smart farming has reduced the adverse impact of conventional agricultural practices, such as the extensive use of fertilizers and pesticides, which often result in soil depletion, water contamination, and loss of biodiversity, ultimately leading to environmental degradation.

**Figure 1:**
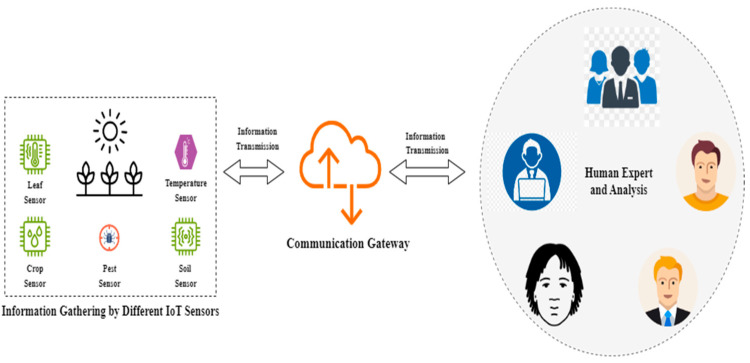
Internet-based framework for smart farming.

Smart farming practices have also effectively mitigated water scarcity, as these systems enable efficient water management through precise irrigation systems that conserve water but enhance crop yield and growth by maintaining ideal soil moisture levels at all times. These advantages thus help improve sustainability and food security by guaranteeing a stable supply of food to meet the demands of the growing population [20]. Conventionally, a smart rural farm works through the interconnected system of devices, known as the IoT, that employs various types of devices to collect data, communication networks to transmit this data, and cloud servers that manage and analyze the collected data through AI–human-centric experts. Figure 1 illustrates such a typical AI and IoT framework under real-time conditions.

As depicted in Figure 1, the importance of smart farming can be observed from the IoT applications in algorithms that help monitor soil, crop growth, and the environment, along with AI algorithms that help analyze the data collected by the IoT devices to predict crop yields, detect diseases, and recommend optimal planting schedules through deep learning models [21].

Prior research discusses the architecture of the IoT for smart farming, which consists of four main layers: perception, transport, processing, and application. The perception layer comprises physical devices like sensors and UAVs that can be custom-built using platforms such as Arduino and Rasberry Pi. The data gathered from these devices is transmitted to the processing layer by the transport layer using various communication protocols like Wi-Fi, GPRS, 3G, Bluetooth, SigFox, LoRaWAN, and ZigBee. While the processing layer handles the data storage, management, and analysis by employing big data technologies, the application layer provides the necessary management information to farmers through mobile and web applications for effective agriculture production.

Combining the IoT with data analytics allows for extracting essential insights from large datasets of collected information. Prior research has shown how big data can enhance farm management practices and optimize the food supply chain. Crop forecasting helps farmers plan, make decisions, and conduct additional studies on the yield quality by predicting the crop yield before the crop harvest. Additionally, maturity sensors monitor the crop at various phases of development, considering elements like fruit color and size to determine the ideal time for harvesting. Multicolor (RGB) satellite photographs are also used to cover large areas for farm monitoring and control. These real-time data are displayed in mobile applications for farmers and other stakeholders by developing and installing a yield monitor. Another recent advancement in smart farming has been the deployment of automatic and remote-controlled mobile robotics that can operate tasks such as planting, weeding, and harvesting, reducing labor costs, increasing efficiency, and enhancing productivity and sustainability.

## 4. Privacy-Centric Concerns in AI and IoT in Smart Farming Monitoring and Control

### 4.1. Analysis of Privacy Risks

#### 4.1.1. Vulnerability in Information

Farming-related AI and IoT devices gather a wide variety of data, such as crop yields, soil conditions, meteorological information, and even personal information about the farmer. This vast amount of collected data, necessary for effective farm monitoring and control, poses a serious risk if improperly secured [22]. Due to its sensitive nature, this data may also be subject to cyberattacks, which could result in monetary losses or harm to one’s reputation. The likelihood of these assaults is also higher in rural areas where cybersecurity precautions are weaker [23]. Furthermore, because AI algorithms can deduce extra sensitive information from seemingly non-sensitive data, the integration of AI in data processing might further complicate privacy and increase the potential effect of a data leak [24].

#### 4.1.2. Potential Hazards of Cloud Storage

The massive volumes of data collected by IoT devices are often managed and stored in cloud storage. Though this provides accessibility and scalability, it also raises concerns like data breaches and illegal access by outside parties [25]. Also, since farmers frequently rely on outside service providers for cloud storage, it raises questions about data handling and security procedures. Privacy problems may also be made worse by the lack of direct control over these cloud servers [26]. Another major worry is the possibility of insider attacks at the cloud service provider, where staff members may access or abuse the data [27].

#### 4.1.3. Interception throughout Transmission

Information sent from IoT devices to cloud servers or other devices may be intercepted, which is particularly troubling in rural locations where secure network infrastructure might not be as developed [28]. Additionally, in rural farming installations, standard encryption protocols are often ignored when transferring data across open or unprotected networks, increasing the risk of being intercepted by unauthorized parties and leading to privacy violations [6,29].

#### 4.1.4. Failure of Data Governance

Data management for farmers can be challenging for those unfamiliar with data collection, storage, and application, as the lack of transparency and control may pose significant privacy risks. Additionally, contracts with technology providers may include opaque terms and conditions on data usage that farmers cannot fully comprehend, leading them to unintentionally consent to extensive data usage rights. Addressing these privacy concerns requires transparent data governance policies that give farmers more control and knowledge over their data [30,31].

### 4.2. Privacy Challenges in Smart Farming

#### 4.2.1. Unauthorized Data Access Incidents

Significant privacy violations occurred when an agri-tech company illegally accessed data from IoT devices used by large-scale farming operations. This particular company engaged in analytic services exploited a weakness in data transmission and collected confidential information without consent [9]. The collected data included comprehensive details on agricultural productivity, soil health, irrigation schedules, and personal information of the farm owners and crew, prompting serious concerns about the protection of personal data and the operational confidentiality of the farm [32]. The consequence of this incident included a legal battle and substantial financial losses for the farming company. This highlights the disastrous impact of inadequate data security protocols in IoT-enabled farming contexts [33].

#### 4.2.2. Cybersecurity Breaches

Another notable incident was a cyberattack on a mid-sized farm’s cloud storage system, exploiting vulnerabilities in the cloud server provider’s security system [34]. Hackers bypassed security measures to access a vast amount of information, including real-time crop conditions, agriculture equipment usage, and financial records [35]. Furthermore, personal information such as addresses and phone numbers were also compromised, disrupting farm operations and causing significant challenges for individuals whose identities were stolen, affecting their privacy.

### 4.3. Strategies for Enhancing Privacy in Smart Farming

#### 4.3.1. Implementing Robust Data Encryption

In rural farming communities where information technology (IT) infrastructure is sometimes lacking, efficient data encryption is crucial to protect data collected through various IoT devices while in use and at rest [7]. Additionally, in remote areas with higher risks of local network breaches, advanced encryption technologies ensure that data remains unintelligible to unauthorized users [36]. Encryption also protects data from internal and external vulnerabilities, preventing unauthorized access by workers and service providers [37].

#### 4.3.2. Ensuring Secure Transmission of Data

Since data transmission from IoT devices to cloud servers is a critical point where information can be intercepted, it is necessary to use reliable transmission mechanisms to prevent unauthorized access [25]. Accordingly, data should be encrypted during transfer through technologies like Secure Sockets Layer and Transport Layer Security (SSL/TLS) since these provide a secure channel over an insecure network. This assures the authenticity and privacy of any data exchanged between servers located in the cloud and IoT devices [6,38].

#### 4.3.3. Enhancing Knowledge and Training

A frequently overlooked aspect of data privacy is human errors that can be mitigated with proper guidance and instruction on the importance of data confidentiality and the risks of information leaks. Providing regular training and updates on emerging privacy issues, fraud attempts, and the best counter practices will also help maintain a knowledgeable workforce capable of securely managing personal information [29,39].

**Figure 2:**
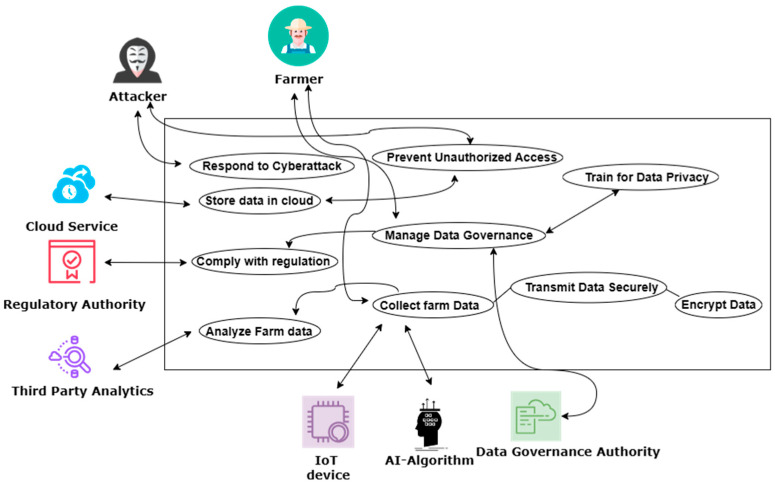
Framework illustrating roles and need for data privacy and security.

The above discussion illustrates how exhaustive the network of connections and protocols must be to ensure data security in the rural farm when employing AI and the IoT for a smart farming framework. This is illustrated in Figure 2, which highlights the critical measures that need to be taken to prevent security attacks and the roles of different stakeholders in a smart rural farm for monitoring and control.

## 5. Proposed Protocol

**Figure 3:**
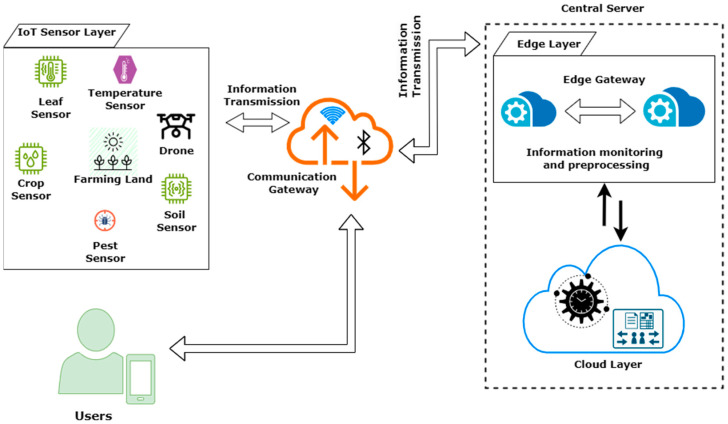
Framework for each entity of the proposed protocol.

The authentication protocol described in this work involves three entities: the user $U_{r_{j}}$, the IoT Sensor layer ${IoT}_{{SL}_{j}}$, and the central server CS. Upon the user’s login to the IoT Sensor layer, the protocol carries out authentication for every entity. Figure 3 depicts this framework for each entity.

The proposed authentication protocol consists of three sequential phases: registration, login, and verification. CS is a trustworthy authentication central server that includes an Edge gateway and cloud layer that is accountable for the user and the IoT sensor layer’s registration and authentication. Furthermore, the proposed verification protocol utilizes timestamps, necessitating the temporal synchronization between the authentication server CS, user $U_{r_{j}}$, and IoT sensor layer ${IoT}_{{SL}_{j}}$. Table 1 below lists the various notations of the proposed protocol and its description. 

### 5.1. Registration Phase

**Figure 4:**
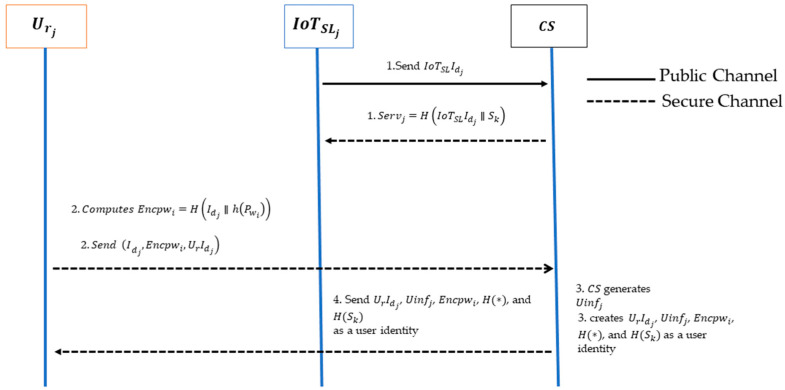
Registration phase.

At the registration phase, the user, $U_{r_{j}}$ and the IoT server, ${IoT}_{{SL}_{j}}$, initiate a registration request to the central server CS. As a reciprocal action, the central server provides the user, $U_{r_{j}}$, with the required values for the login and authentication stages to the IoT server, ${IoT}_{{SL}_{j}}$, as depicted in Figure 4.

${IoT}_{{SL}_{j}}$ transmits its identity value, ${IoT}_{SL}I_{d_{j}}$, to CS through a secure channel. CS then calculates the ${Serv}_{j}$ (Server information) value, which contains the information sent to ${IoT}_{{SL}_{j}}$ by the IoT server through a secure connection.

$${Serv}_{j}=H({IoT}_{SL}I_{d_{j}}∥S_{k})$$

- The $U_{r_{j}}$ selects the user ID, $I_{d_{j}}$, and password, $P_{w_{i}}$, calculates the encrypted password, ${Encpw}_{i}$, and transmits the registration request message ${(I}_{d_{j}},{Encpw}_{i},{U_{r}I}_{d_{j}})$ together with the user’s anonymity values to the central server CS.
  $${Encpw}_{i}=H(I_{d_{j}}∥h(P_{w_{i}}))$$
- The CS generates the user’s confidential information value, ${Uinf}_{j}$, and creates ${U_{r}I}_{d_{j}}$, ${Uinf}_{j}$, ${Encpw}_{i}$, H(∗), and $H(S_{k})$ as a user identity.
  $${Uinf}_{j}=H({Encpw}_{i}∥S_{k})$$
- The user’s anonymity value, ${U_{r}I}_{d_{j}}$, the user’s secret information value, ${Uinf}_{j}$, and the status-bit values are all stored in CS. The status-bit value is stored as 1 if the user completes the registration process, and 0 if there is no registration. Then CS issues an identity to $U_{r_{j}}.$

### 5.2. Login and Verification Phases

**Figure 5:**
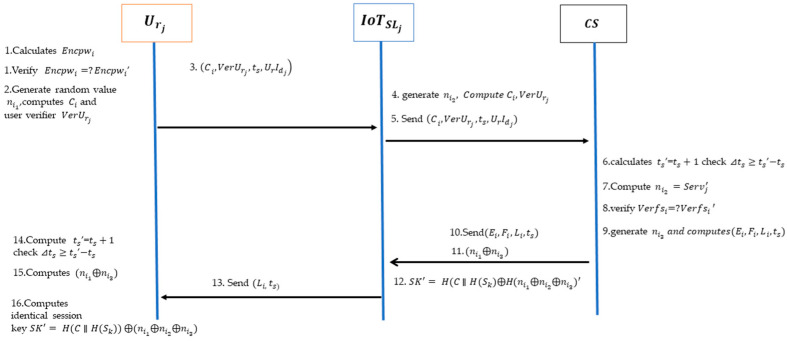
Login and verification phases.

The verification of legitimate users is conducted during the login and verification phase as shown in Figure 5. The user, $U_{r_{j}}$, sends a registration request message to the IoT sensor layer, ${IoT}_{{SL}_{j}}$, in order to log in, and CS verifies each entity. Then, $U_{r_{j}}$, ${IoT}_{{SL}_{j}}$, and CS generates the similar session key.

- $U_{r_{j}}$ inputs their ${U_{r}I}_{d_{j}}$ and password, $P_{w_{i}}$. CS calculates the ${Encpw}_{i}$′ and compares the information with ${Encpw}_{i}$. The user is confirmed as a legitimate user if the information matches. Termination of the session occurs when the information fails to correspond.
  $${Encpw}_{i}\prime =H(I_{d_{j}}∥h(P_{w_{i}}))$$
  $${Encpw}_{i}{=?Encpw}_{i}\prime$$
- $U_{r_{j}}$, the verified user, selects a random value, $n_{i_{1}}$, for each session. Using $H(S_{k})$, ${Uinf}_{j}$, and the chosen random value $n_{i_{1}}$ computes $C_{i}$ and the user verifier $VerU_{r_{j}}$. Next, a timestamp called $t_{s}$ is generated.
  $$C_{i}={Uinf}_{j}⨁\;H(S_{k})⨁n_{i_{1}}$$
  $$VerU_{r_{j}}=H(H(S_{k})∥n_{i_{1}})$$
- The user, $U_{r_{j}}$, sets up the login request message ${(C}_{i},VerU_{r_{j}},t_{s},{U_{r}I}_{d_{j}})$ by including their anonymity value, ${U_{r}I}_{d_{j}}$, calculating $C_{i}$ and $t_{s}$, and then transmits the message to the IoT sensor layer, ${IoT}_{{SL}_{j}}$.
- The IoT sensor layer, ${IoT}_{{SL}_{j}}$, upon receiving the login request message from $U_{r_{j}}$, chooses a random number, $n_{i_{2}}$, for each session and calculates $D_{i}$ and ${Verfs}_{i}$ using the ${Serv}_{j}$ value received during the registration step.
  $$D_{i}={Serv}_{j}⨁n_{i_{2}}$$
  $${Verfs}_{i}=H(H({IoT}_{SL}I_{d_{j}}∥S_{k})∥n_{i_{2}})$$
- ${IoT}_{{SL}_{j}}$ sends the login request message to CS. The message is set up for the $C_{i}$, ${U_{r}I}_{d_{j}}$ (received from the user $U_{r_{j}}$), the ${IoT}_{{SL}_{j}}$ unique identification value ${IoT}_{SL}I_{d_{j}}$, $D_{i}$ (which was generated earlier), and the timestamp $t_{s}$.
- The CS that received the login request message from ${IoT}_{{SL}_{j}}$ calculates $t_{s}$′ = $t_{s}+1$, and then verifies the difference between $t_{s}$′ and $t_{s}$, denoted as $⊿t_{s}\geq$ $t_{s}$′$-t_{s}$. In this context, $⊿t_{s}$′ represents the timestamp indicating the moment the server received the login message. $t_{s}$ refers to the shortest possible authentication time, taking into account the time it takes for the login message to be transmitted.
- CS produces ${Serv}_{j}$ using the received ${IoT}_{SL}I_{d_{j}}$ value and its own master key, and then retrieves the $n_{i_{2}}$ value using the $D_{i}$ value obtained from the login request message.
  $${Serv}_{j}^{\prime }=H({IoT}_{SL}I_{d_{j}}∥\;S_{k})$$
  $$n_{i_{2}}^{\prime }={Serv}_{j}^{\prime }\;⨁D_{i}$$
- By utilizing the calculated $n_{i_{2}}$ value, CS generates the ${Verfs}_{i}$Z value. If the ${Verfs}_{i}$′ value matches the $D_{i}$ value received in the login request message, it is confirmed as the legitimate ${IoT}_{{SL}_{j}}$. If there is no match, the connection is terminated.
  $${Verfs}_{i}\prime =H(H({IoT}_{SL}I_{d_{j}}∥S_{k})∥n_{i_{2}}\prime )$$
  $${Verfs}_{i}={?Verfs}_{i}\prime$$
- Using the ${U_{r}I}_{d_{j}}$ from the login request message, the system can search for the ${U_{r}Info}_{i}$ generated during the registration phase. CS randomly selects the value $n_{i_{3}}$ and calculates the $n_{i_{1}}\prime$ value by using the received $C_{i}$ value, the generated $H(S_{k})$, and the previously retrieved ${U_{r}Info}_{i}$. By utilizing a calculated $n_{i_{1}}$ value and $H(S_{k})$, the $VerU_{r_{j}}$′ value is generated. After verifying the received $VerU_{r_{j}}$ value with the login request message, the system authenticates the user as legitimate and generates the session key SK. If there is no match, the session will be terminated.
  $$n_{i_{1}}^{\prime }={U_{r}Info}_{i}⨁H(S_{k})⨁C_{i}$$
  $$VerU_{r_{j}}^{\prime }=H(H{(S}_{k})∥n_{i_{1}}\prime )$$
  $$VerU_{r_{j}}=?VerU_{r_{j}}\prime$$
  $${SK}_{i}=H(C∥H{(S}_{k})⨁H(n_{i_{1}}⨁n_{i_{2}}⨁n_{i_{3}}))$$
- A timestamp, $t_{s}$, is generated afterward, it calculates the $E_{i},F_{i},L_{i}$, and transmits the mutual authentication message $\left(E_{i},F_{i},L_{i},t_{s}\right)$ to ${IoT}_{{SL}_{j}}$.
  $$E_{i}=n_{i_{1}}⨁n_{i_{3}}⨁H({IoT}_{SL}I_{d_{j}}⨁n_{i_{2}})$$
  $$F_{i}=H(C∥H{(S}_{k})⨁H⨁n_{i_{2}})$$
  $$L_{i}=n_{i_{1}}⨁n_{i_{3}}⨁H(C∥H{(S}_{k})$$
- Upon receiving the mutual authentication message, ${IoT}_{{SL}_{j}}$ calculates the $n_{i_{1}}⨁n_{i_{3}}$′ using its own ${IoT}_{SL}I_{d_{j}}$ value and the random value $n_{i_{2}}$.
  $${\left(n_{i_{1}}⨁n_{i_{3}}\right)}^{\prime }=E_{i}⨁H({IoT}_{SL}I_{d_{j}}⨁n_{i_{2}})$$
- This computes the value of $H(C∥H{(S}_{k})$′ by utilizing its own ${IoT}_{SL}I_{d_{j}}$ value and the random value $n_{i_{2}}$, with the $F_{i}$ value obtained from the mutual authentication message. It generates the session key, SK, by combining its own random value, $n_{i_{2}}$, with the previously computed $\left(n_{i_{1}}⨁n_{i_{3}}\right)$′. Afterwards, the ${IoT}_{{SL}_{j}}$ calculates $L_{i}$ and sends a login response message ${(F}_{i},t_{s})$ to the user $U_{r_{j}}$.
  $$H{\left(C∥H{(S}_{k}\right)}^{\prime }=F_{i}⨁H({IoT}_{SL}I_{d_{j}}⨁n_{i_{2}})$$
  $$SK^{\prime }=H(C∥H{(S}_{k})⨁H(n_{i_{1}}⨁n_{i_{2}}⨁n_{i_{3}})\prime$$
  $$L_{i}={(n}_{i_{2}}⨁n_{i_{3}})⨁H(C∥H{(S}_{k})$$
- Upon receiving the login request message, the user performs a computation to confirm the time difference meets the required criteria. $t_{s}$′ represents the timestamp at which the server receives the login message, while $⊿t_{s}\geq$ $t_{s}$′$-t_{s}$ represents the minimum authentication time, taking into account the transmission time for the login message. Using the $L_{i}$ value obtained from the mutual authentication message, $U_{r_{j}}$ is able to calculate the value of ${(n}_{i_{2}}⨁n_{i_{3}})$ using the $C_{i}$ value provided by the user and the $H{(S}_{k})$ value.
  $${(n}_{i_{2}}⨁n_{i_{3}})\prime =L_{i}⨁H(C∥H{(S}_{k}))$$
- The user, $U_{r_{j}}$, can independently process a randomly generated value ${(n}_{i_{1}})$ along with ${(n}_{i_{2}}⨁n_{i_{3}})$. By utilizing its own value $C_{i}$ and the $H{(S}_{k})$ value, the $U_{r_{j}}$ can generate the session key SK. Thus, the user $U_{r_{j}}$, ${IoT}_{{SL}_{j}}$, and authentication server CS can authenticate by producing an identical session key.
  $$SK^{\prime }=H(C∥H{(S}_{k}))\;⨁{(n_{i_{1}}⨁n}_{i_{2}}⨁n_{i_{3}})$$

## 6. Formal Security Analysis and Verification Using the AVISPA Tool

**Figure 6:**
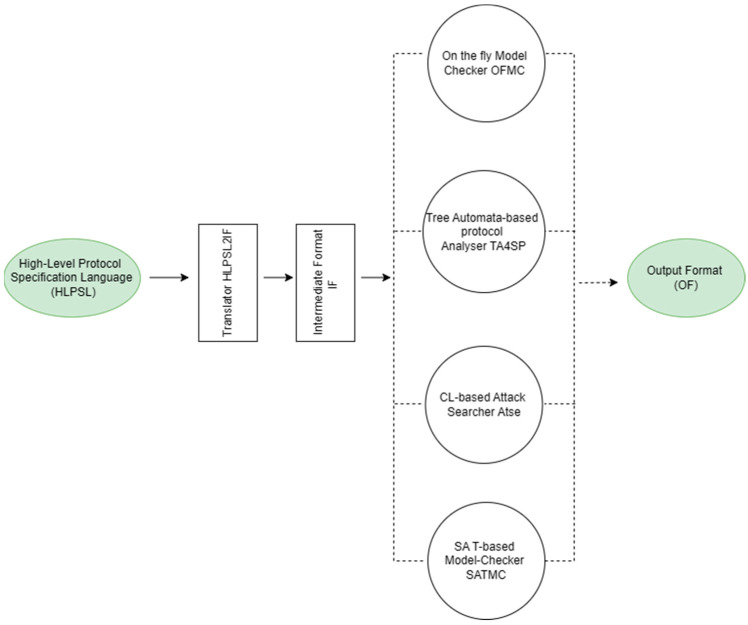
Architecture of the AVISPA tool.

The robustness of the proposed protocol is tested using the AVISPA tool, which employs a role-based scripting language, High-Level Protocol Specification Language (HLPSL) that helps with protocol analysis and implementation to determine whether a security protocol is SAFE or UNSAFE [40]. AVISPA mimics the protocol behavior to find potential vulnerabilities using back-ends such as the OFMC (on-the-fly model checker), CL-AtSe (constraint-logic-based attack searcher), SATMC (SAT-based model checker), and TA4SP (tree automata-based security protocol), shown in Figure 6, of which the OFMC and CL-AtSe back-ends were used to simulate our proposed protocol. Also, the Dolev–Yao threat model was implemented to examine the presence of intruder attacks in the protocol [41].

**Figure 7:**
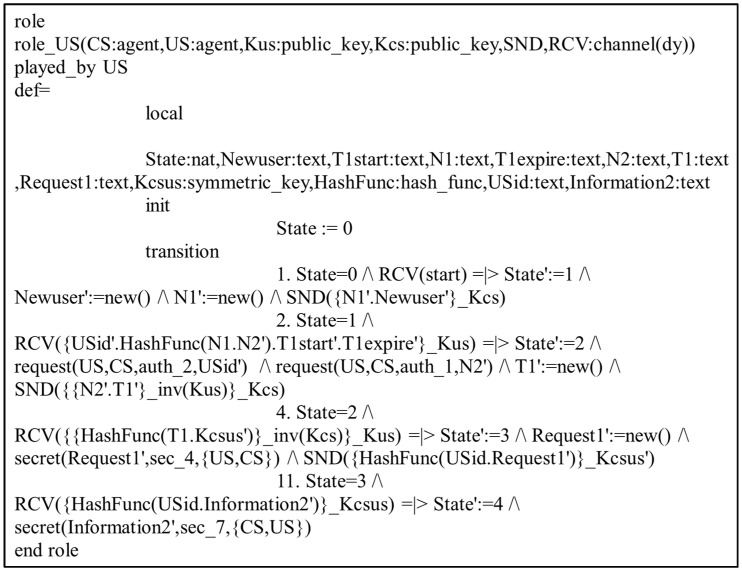
Role specification of the User (US) in HLPSL.

**Figure 8:**
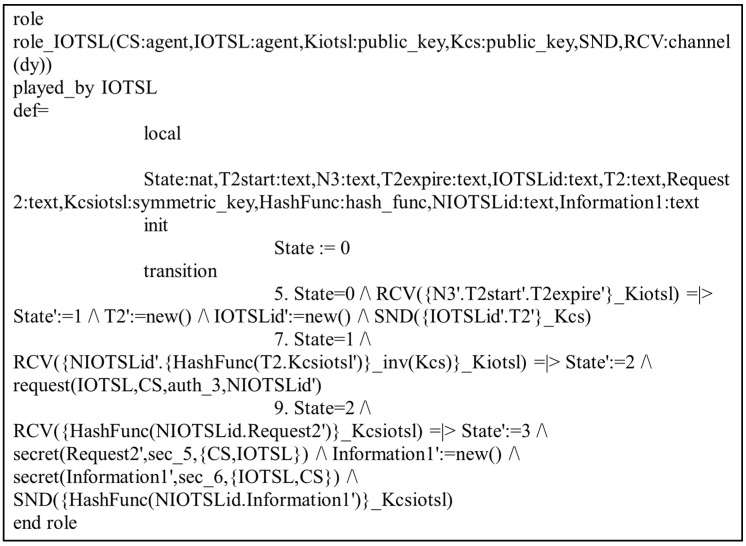
Role specification of the IoT Sensor Layer (IOTSL) in HLPSL.

**Figure 9:**
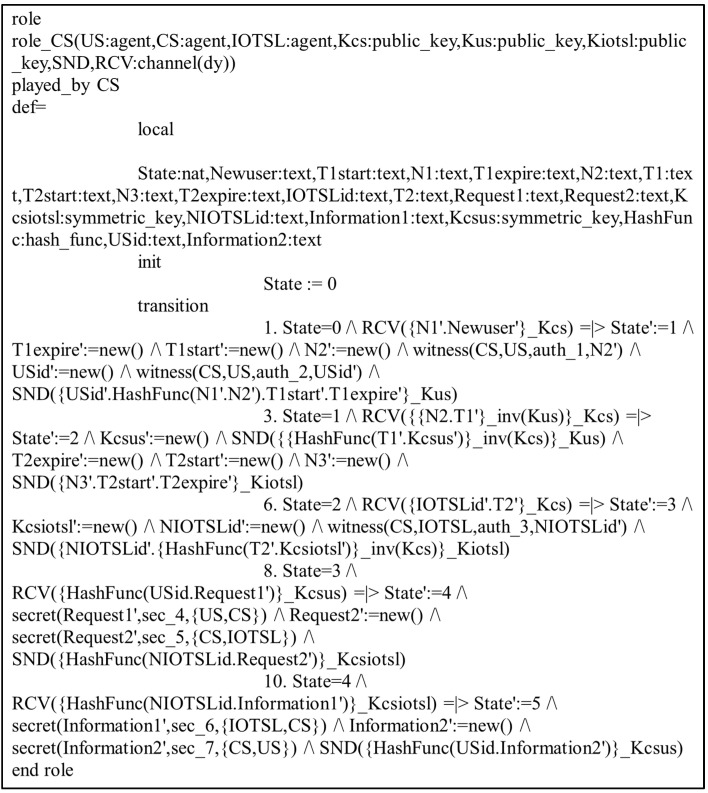
Role specification of the Central Server (CS) in HLPSL.

**Figure 10:**
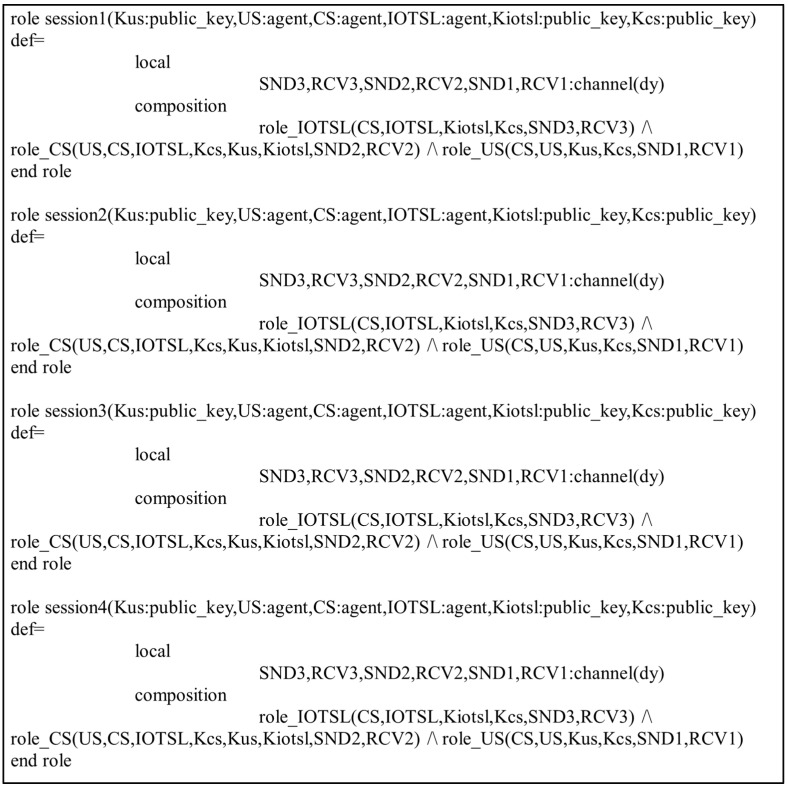
Role specification of sessions in AVISPA.

**Figure 11:**
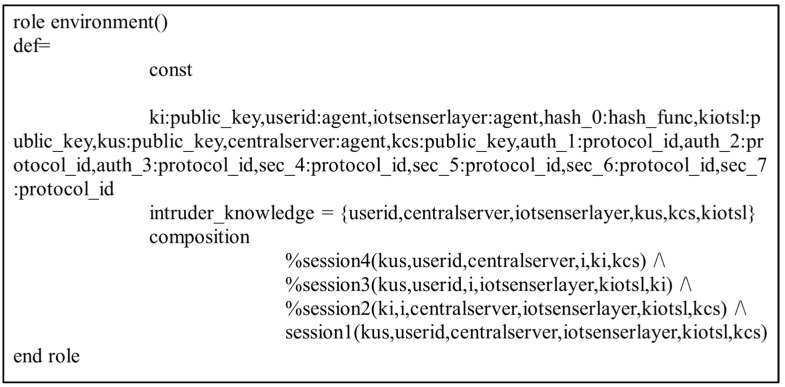
Role specification of the environment in AVISPA.

**Figure 12:**
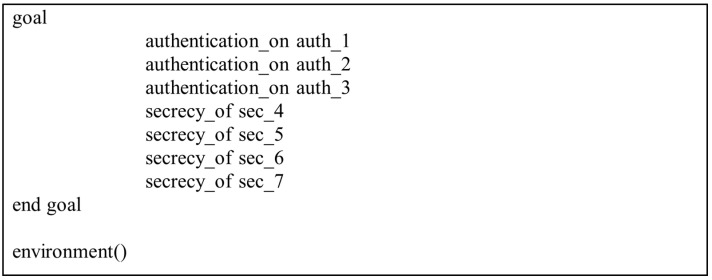
Role specification of goals in AVISPA.

**Figure 13:**
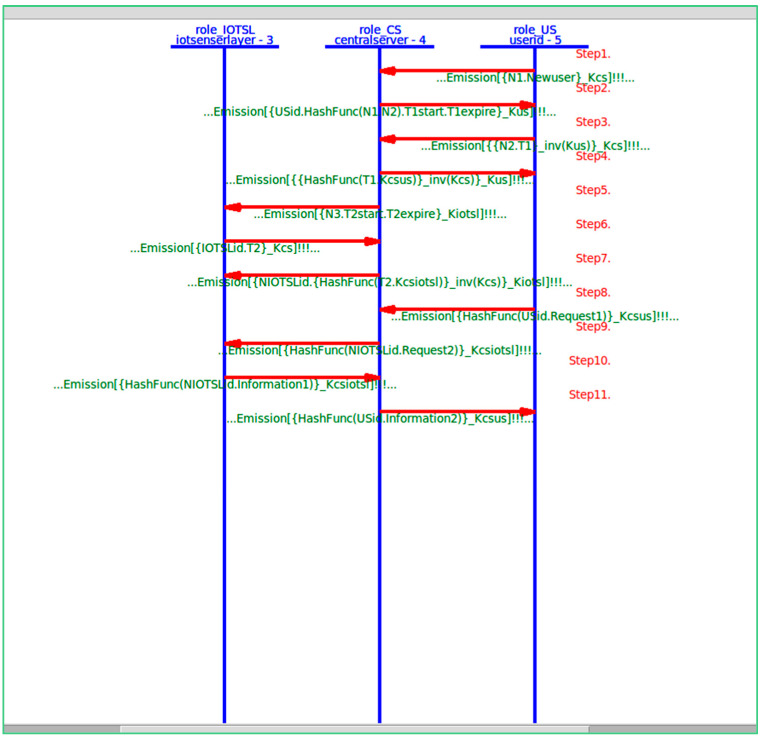
The message sequence chart from the protocol simulation.

In the present study, the different entities, User, IoT Senser Layer, and Central Server, are represented as the agents US, IOTSL, and CS, respectively. Figure 7, Figure 8 and Figure 9 show the HLPSL script and their roles, while Figure 10, Figure 11 and Figure 12 discuss the HLPSL script of the sessions, environment, and goals. Figure 13 shows the SPAN protocol simulation built using the message sequence between the agents discussed in the next section.

### 6.1. Protocol Steps and Messages

1. US->CS: /\ SND({N1’.Newuser’}_Kcs)

Description: The User (US) initiates the protocol by registering itself with the identity ‘Newuser’ and sending its identity along with a nonce ‘N1’ encrypted with the public key of Central Server ‘Kcs’.

Purpose: To securely communicate the new user’s identity and nonce to the Central Server for registration.

2. CS->US: /\ SND({USid’.HashFunc(N1’.N2’).T1start’.T1expire’}_Kus)

Description: The Central Server (CS) decrypts the previous encrypted message using its private key. After verifying the user, it assigns an identity ‘Usid’ and sends it along with a hash function containing the concatenation of nonces ‘N1’ and ‘N2’, and time stamps ‘T1start’ and ‘T1 expire’, all encrypted using the public key of User ‘Kus’.

Purpose: To provide the user with an identity, the authentication of nonces, and the time stamps of the session while ensuring integrity.

3. US->CS: /\ SND({{N2’.T1’}_inv(Kus)}_Kcs)

Description: The User (US) decrypts the received message using its private key and sends back the nonce ‘N2’ that is computed from the hash function, along with token ‘T1’, which are encrypted with its private key ‘Kus” and then encrypted again with the public key of Central Server ‘Kcs’.

Purpose: To confirm the user’s identity and authentication of the nonce, ensuring mutual authentication and integrity through the asymmetric key pair providing a signature.

4. CS->US: /\ SND({{HashFunc(T1’.Kcsus’)}_inv(Kcs)}_Kus)

Description: The Central Server (CS) decrypts the received encrypted message and responds by hashing the concatenation of the received token ‘T1’ with a newly generated session key ‘Kcsus’. This hash is encrypted with the private key from the Control Server ‘Kcs” and then with the public key from ‘Kus’.

Purpose: To provide a session key and ensure the authentication, integrity, and confidentiality of the communication.

5. CS->IOTSL: /\ SND({N3’.T2start’.T2expire’}_Kiotsl)

Description: The Central Server (CS) initiates communication with the IoT Sensor Layer (IOTSL) by sending a nonce ‘N3’ and the time stamps ‘T2start’ and ‘T2expire’ encrypted with the public key from IOTSL ‘Kiotsl’.

Purpose: To securely establish a session with the IoT Sensor Layer.

6. IOTSL->CS: /\ SND({IOTSLid’.T2’}_Kcs)

Description: After decrypting the message using its private key, the IoT Sensor Layer (IOTSL) sends its identity ‘IOTSLid’ and a token ‘T2’ encrypted with the public key from the Central Server ‘Kcs’.

Purpose: To confirm its identity and establish a session with the Central Server.

7. CS->IOTSL: /\ SND({HashFunc(NIOTSLid.Request2’)}_Kcsiotsl)

Discussion: The Central Server (CS) decrypts the previous message and, after verifying the IoT device, sends its identity ‘NIOTSLid’ and a hash function containing the concatenation of token ‘T2’ and the newly generated session key ‘Kcsiotsl’, encrypted with the private key from the Central Server ‘Kcs” and the public key from the IoT Sensor Layer ‘Kiotsl’.

Purpose: To confirm and authenticate the IoT Sensor Layer and establish a secure session key.

8. US->CS: /\ SND({HashFunc(USid.Request1’)}_Kcsus’)

Description: The User (US) sends a request ‘Request 1’ in a hash concatenation of the user identity ‘Usid’, encrypted with the session key ‘Kcsus’.

Purpose: To securely send a request to the Central Server, ensuring fast and secure transmission of bulk data.

9. CS->IOTSL: /\ SND({HashFunc(NIOTSLid.Request2’)}_Kcsiotsl)

Description: Following the user’s request, the Central Server (CS) sends the computed request ‘Request 2’ concatenated with ‘NIOTSLid’ and the assigned identity of the IoT device encrypted with the session key ‘Kcsiotsl’.

Purpose: To securely relay the user’s request to the IoT Senser Layer.

10. IOTSL->CS: /\ SND({HashFunc(NIOTSLid.Information1’)}_Kcsiotsl)

Description: The IoT Sensor Layer (IOTSL) decrypts the message and sends the requested information ‘Information 1′ concatenated with its assigned identity ‘NIOTSLid’ encrypted with the session key ‘Kcsiotsl’.

Purpose: To securely send the requested information back to the Central Server.

11. CS->US: /\ SND({HashFunc(USid.Information2’)}_Kcsus)

Description: After decrypting the message from the IoT Sensor Layer, the Central Server (CS) sends the newly computed information ‘Information2’ concatenated with the assigned user identity ‘Usid’ encrypted with the session key ‘Kcsus’.

Purpose: To securely deliver the requested information back to the user.

### 6.2. Secrecy and Authentication Goals

CS authenticates US on N2: The Central Server (CS) requests the authentication of ‘N2’ on User US through /\ witness(CS,US,auth_1,N2’) and /\ request(US,CS,auth_1,N2’).

CS authenticates US on Usid: The Central Server (CS) requests the authentication of USid on User US through /\ witness(CS,US,auth_2,USid’) and /\ request(US,CS,auth_2,USid’).

CS authenticates IOTSL on NIOTSLid: The Central Server (CS) requests the authentication of NIOTSLid on the IoT Sensor Layer through /\ witness(CS,IOTSL,auth_3,NIOTSLid’) and /\ request(IOTSL,CS,auth_3,NIOTSLid’).

secrecy_of Request1 [US,CS]: Secrecy of message Request1 between the User US and Central Server (CS) /\ secret(Request1’,sec_4,{US,CS}).

secrecy_of Request2 [CS,IOTSL]: The secrecy of message Request2 between the Central Server (CS) and the IoT Sensor Layer (IOTSL) /\ secret(Request2’,sec_5,{CS,IOTSL}).

secrecy_of Information1 [IOTSL,CS]: The secrecy of message Information1 between the IoT Sensor Layer (IOTSL) and Central Server (CS) /\ secret(Request2’,sec_5,{CS,IOTSL}).

secrecy_of Information2 [CS,US]: The secrecy of message Information2 between Central Server (CS) and User US /\ secret(Information2’,sec_7,{CS,US}).

### 6.3. Attack Prevention

Several security issues are addressed by the proposed protocol, which are discussed below.

Identity and Password Guessing Attack: In this type of attack, the intruder attempts to guess a user’s identity or password by trying multiple combinations, especially if it uses a low-entropy password or an easily guessable identity. Our proposed protocol ensures that this is not possible through the use of cryptographic hashing functions and hybrid encryption. To understand the identity of the User, the intruder will have to guess multiple unknown parameters simultaneously, all of which are protected by non-invertible hash functions.

Impersonation-of-user attack: In this type of attack, the attacker poses as the one who initiated the communication. Our proposed protocol mitigates this by involving nonces (N1 and N2) and verification through the hash function, making it difficult for the attacker to use old messages or predict values needed to impersonate the user. For instance, /\({N1′. Newuser’}_Kcs) and /\({USid’.HashFunc(N1′.N2′).T1start’.T1expire’}_Kus) involve the fresh exchange of nonces, ensuring message uniqueness and validity for only one session.

Impersonation-of-server attack: This attack occurs when the attacker tries to impersonate the server to intercept or alter communications. This is prevented by requiring mutual authentication and the use of encrypted nonces. Messages encrypted with the public key ensure that only the legitimate server can decrypt and respond correctly: /\ ({USid’.HashFunc(N1′.N2′).T1start’.T1expire’}_Kus). The use of the witness function, also /\ witness(CS,US,auth_1,N2′), verifies the authenticities of the nonces and the communication parties, preventing server impersonation.

Privilege Insider Attack: This attack occurs when a malicious insider attempts to misuse their access to gain unauthorized information. Our protocol limits such attacks by ensuring that sensitive information is not directly accessible or stored in a decipherable form. For instance, identities and other data are hashed before storage or transmission, and the function /\ ({HashFunc(USid.Request1′)}_Kcsus’) /\ ({HashFunc(NIOTSLid.Information1′)}_Kcsiotsl) ensures that even if an insider accesses stored data, they cannot easily reverse-engineer to compute the original data.

Replay attacks: Replay attacks are possible when the intruder is able to send an old message and gain unauthorized access successfully. The proposed protocol withstands these attacks using fresh nonces and current time stamps, and it generates /\ T1start’:=new() /\ T1expire’:=new() /\ N1’:=new(). Since new() operators are performed at every stage of the communication, it prevents the intruder from performing a replay attack (/\ T2start’:=new() /\ T2expire’:=new() /\ N2’:=new()). Four protocol sessions were considered for testing against a replay attack, where one session contained all legitimate participants, and all other sessions contained an intruder impersonating any of the legitimate participants. Across all configurations, with different attack scenarios, the results showed that the protocol is safe using both OFMC and CL-AtSe.

Man-in-the-middle attack: In this particular type of attack, the intruder can intercept, eavesdrop, or alter communication messages. The proposed protocol withstands this by ensuring all communication uses asymmetric key pairs for encryption through signatures and hash functions. So, any attempt to alter the communication would be detected by the mismatch in the computed hash values.

Anonymity and untraceability attack: In these types of attacks, the intruder tries to determine the agent’s identity from the messages, which is impossible in the proposed protocol as it initiates communication using random nonces. Also, authentication messages are encrypted with random session keys that are generated for each session, which are secure through hash functions and asymmetric key pair encryption.

Parallel attacks: The proposed protocol withstands these types of coordinated multi-attacks performed simultaneously by enforcing sequential steps that require exchanging information and credentials at each stage. Using authentication goals also prevents the intruder from successfully impersonating multiple roles.

Furthermore, the use of encryption and decryption functions, symmetric keys, hash functions, and concatenation ensures the security of the proposed protocol. Additionally, the protocol incorporates the use of hybrid encryption, which involves the concept of using both asymmetric key pairs and symmetric encryption. The asymmetric key pair is used for authentication and facilitating a key exchange of symmetric keys that can be used for bulk data encryption.

### 6.4. Simulation Results

**Figure 14:**
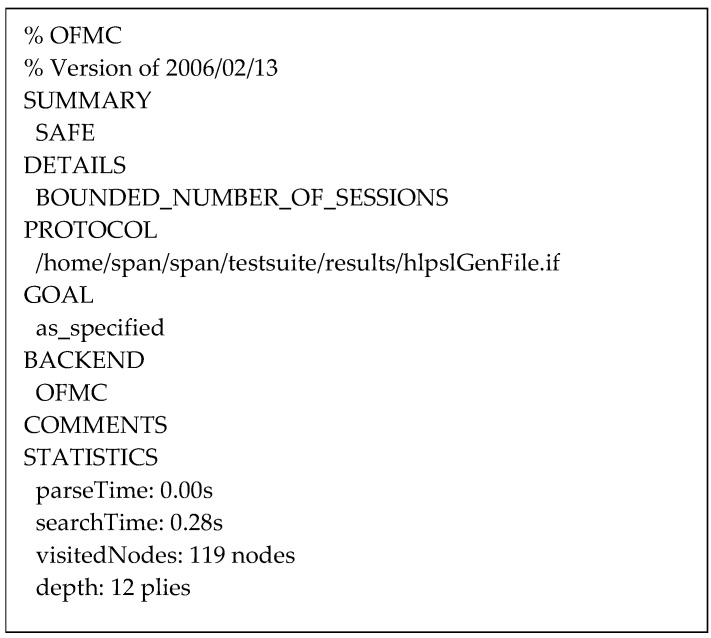
Summary of the protocol simulation using the OFMC back-end.

**Figure 15:**
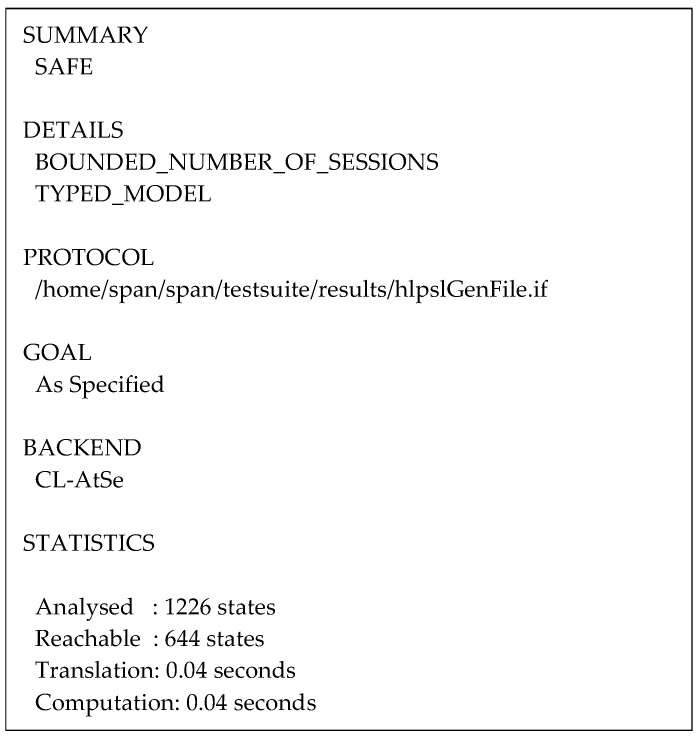
Summary of the protocol simulation using the CL-AtSe back-end.

According to the result from the OFMC back-end depicted in Figure 14, 119 nodes were visited with a search time of 0.28 s and a depth of 12 plies. The protocol analysis using the CL-AtSe back-end shown in Figure 15, reveals that 644 states were reachable from 1226 states with a translation time of 0.04 s and a computation time of 0.04 s.

The obtained summaries demonstrate that the protocol is safe in all sessions without intruding attacks while guaranteeing that all secrecy and authentication goals are fulfilled.

### 6.5. AVISPA Statistical Comparison

The OFMC and Cl-AtSe outcome summaries were compared against various other authentication protocols and are detailed in Table 2. In the table, computation time refers to the complete execution time of a protocol transaction or the length of a series of transactions that are being examined. This can include aspects like the time needed for authentication, key exchange, and data transfer. Since extended transaction durations might not be ideal for applications that need to be completed quickly, it is important to know the computation time to assess its practical applicability. In terms of performance testing comparisons, our proposed protocol was simulated several times using an Intel Core i5 Asus computer with 8 GB of RAM and MS Windows 10 Enterprise 64-bit.

The other columns in the table refer to depth and visited nodes. While depth indicates how thoroughly a given scenario is examined, visited nodes refer to how many nodes have been looked into. So, a high number of visited nodes and significant depth suggest that a detailed search was performed quickly.

**Figure 16:**
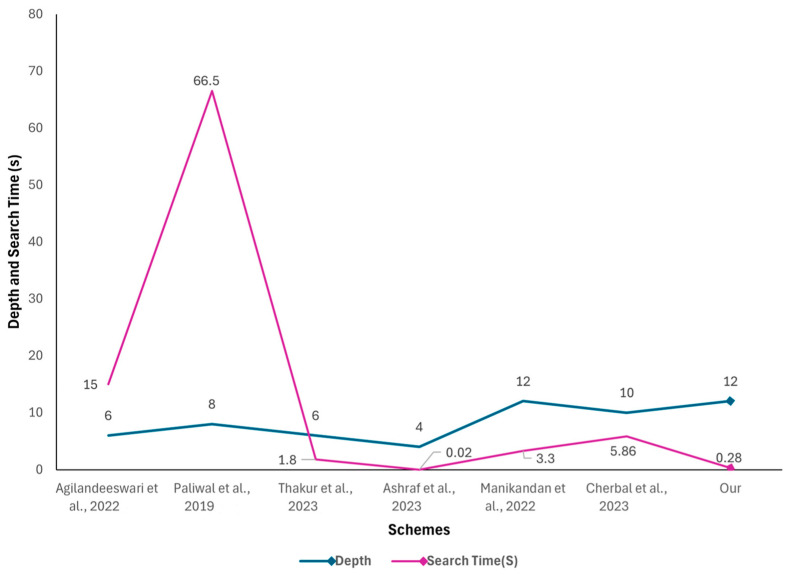
Depth of transaction and Search time [42,43,44,45,46,47].

**Figure 17:**
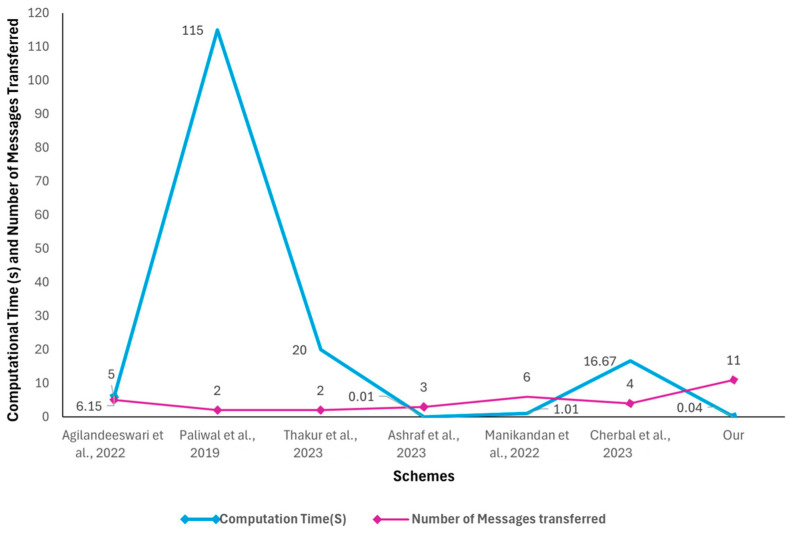
Computational cost and number of messages transferred [42,43,44,45,46,47].

When compared against other protocols, our protocol demonstrates notable improvements in various performance metrics. Firstly, as shown in Figure 16, our protocol matches the depth of [46] with a depth of 12 but with a decrease in the number of visited nodes (119) when compared against [46]’s 348 visited nodes, which demonstrates a more efficient traversal, indicating that our proposal is optimized to reach the desired outcomes and decision-making process within the IoT network. Secondly, regarding the search time, our protocol has one of the fastest search times, second only to [45] which has a much lower depth of four and fewer visited nodes at sixteen. This demonstrates that our protocol is highly efficient in quickly locating the necessary data, which is crucial in real-time applications like smart farming. Thirdly, in terms of computation time, an important parameter for IoT applications that require fast processing and quick response times, our protocol had one of the shortest amounts of time required for computing. Finally, considering the computation time and the ability to handle messages, our protocol could handle the highest number of messages (11) without compromising performance as illustrated in Figure 17. As discussed in the previous section, the higher number of messages has contributed significantly to our protocol’s robust communication and data exchange, which are vital components for comprehensive IoT systems in smart farming.

## 7. Conclusions

The article presents a comprehensive framework for privacy-centered AI and IoT solutions designed to monitor and control smart rural farms. It also presents significant privacy and security concerns by discussing the need for such a framework to meet the increasing demand for agriculture production and the increasing integration of IoT technologies for enhancing efficiency, productivity, and sustainability. To address these concerns, we propose a protocol that ensures secure data exchange across the IoT Sensor Layer, the Central Server, and the User. Through a three-phase scheme, the protocol employs encryption using symmetric and asymmetric key pairs, secure communication channels, concatenations, hash functions, and other rigorous verification mechanisms to efficiently safeguard the data gathered from IoT devices and sensitive information from unauthorized access and potential breaches.

The robustness and efficiency of the proposed protocol were also validated using the AVISPA tool through the OFMC and CL-AtSe back-ends. The simulation finding showed that the proposed protocol can withstand identity guessing, impersonation, replay attacks, man-in-the-middle attacks, safeguard the data from privilege-insider attacks, and ensure anonymity and untraceability. Furthermore, the proposed protocol outperformed existing security protocols in performance metrics, such as computation time, search time, visited nodes, and depth, demonstrating its suitability for real-time applications in smart rural farms. The present study thus adds to the growing body of literature on smart farming by providing a secure and privacy-centric solution to enhance the resilience of rural farm monitoring and control. This study also presents the groundwork for future work to expand on this robust protocol by investigating the application of the protocol in diverse agricultural environments, which will help ensure the protocol’s adaptability and effectiveness in various settings. Additionally, the protocol can be incorporated into large-scale farm operations to examine its scalability and efficiency when handling vast amounts of data and numerous IoT devices. Also, further studies can explore the integration of ML into the protocol to enhance its capabilities for real-time anomaly detection and predictive analytics.

## Figures and Tables

**Table 1:** Proposed protocol notation and description.

Notation	Description
$U_{r_{j}}$	*j* th Users
${IoT}_{{SL}_{j}}$	*j* th IoT Sensors Layer
CS	Central Server
$I_{d_{j}}$	Identity of $U_{r_{j}}$
$P_{w_{i}}$	Password of $U_{r_{j}}$
${U_{r}I}_{d_{j}}$	Unknown value of $U_{r_{j}}$
${IoT}_{SL}I_{d_{j}}$	Identity of ${IoT}_{{SL}_{j}}$
$S_{k}$	Master secret key by CS
$t_{s}$	Timestamp
$n_{i_{1}}$	Random number generated by $U_{r_{j}}$
$n_{i_{2}}$	Random number generated by ${IoT}_{{SL}_{j}}$
$n_{i_{3}}$	Random number generated by CS
SK	session key shared with $U_{r_{j}}$, ${IoT}_{{SL}_{j}}$, CS
H(∗)	One-way hash Function
⨁	XOR Function
∥	Concatenation

**Table 2:** Statistical Comparison of AVISPA, OFMC, and CL-AtSe summaries.

Scheme	Depth	Visited Nodes	Search Time (s)	Computation Time (s)	Number of Messages Transferred
Agilandeeswari et al., 2022 [42]	6	1074	15.0	6.15	5
Paliwal et al., 2019 [43]	8	3425	66.50	115	2
Thakur et al., 2023 [44]	6	128	1.80	20	2
Ashraf et al., 2023 [45]	4	16	0.02	0.01	3
Manikandan et al., 2022 [46]	12	348	3.30	1.013	6
Cherbal et al., 2023 [47]	10	930	5.86	16.67	4
Our	12	119	0.28	0.04	11

## Data Availability

The original contributions presented in the study are included in the article, further inquiries can be directed to the corresponding authors.

## References

[1] Yuan G.N., Marquez G.P.B., Deng H., Iu A., Fabella M., Salonga R.B., Ashardiono F., Cartagena J.A. (2022). A review on urban agriculture: Technology, socio-economy, and policy. Heliyon.

[2] Javaid M., Haleem A., Singh R.P., Suman R. (2022). Enhancing smart farming through the applications of Agriculture 4.0 technologies. Int. J. Intell. Netw..

[3] Mohamed E.S., Belal A.A., Abd-Elmabod S.K., El-Shirbeny M.A., Gad A., Zahran M.B. (2021). Smart farming for improving agricultural management. Egypt. J. Remote Sens. Space Sci..

[4] Saban M., Bekkour M., Amdaouch I., El Gueri J., Ahmed B.A., Chaari M.Z., Ruiz-Alzola J., Rosado-Muñoz A., Aghzout O. (2023). A Smart Agricultural System Based on PLC and a Cloud Computing Web Application Using LoRa and LoRaWan. Sensors.

[5] Zhai Z., Martínez J.F., Beltran V., Martínez N.L. (2020). Decision support systems for agriculture 4.0: Survey and challenges. Comput. Electron. Agric..

[6] Tariq U., Ahmed I., Bashir A.K., Shaukat K. (2023). A Critical Cybersecurity Analysis and Future Research Directions for the Internet of Things: A Comprehensive Review. Sensors.

[7] Aliyu A.A., Liu J. (2023). Blockchain-Based Smart Farm Security Framework for the Internet of Things. Sensors.

[8] Saba T., Rehman A., Haseeb K., Bahaj S.A., Lloret J. (2023). Trust-based decentralized blockchain system with machine learning using Internet of agriculture things. Comput. Electr. Eng..

[9] Gerodimos A., Maglaras L., Ferrag M.A., Ayres N., Kantzavelou I. (2023). IoT: Communication protocols and security threats. Internet Things Cyber-Phys. Syst..

[10] Alqahtani H., Kumar G. (2024). Machine learning for enhancing transportation security: A comprehensive analysis of electric and flying vehicle systems. Eng. Appl. Artif. Intell..

[11] Amiri-Zarandi M., Dara R.A., Duncan E., Fraser E.D.G. (2022). Big Data Privacy in Smart Farming: A Review. Sustainability.

[12] Balaska V., Adamidou Z., Vryzas Z., Gasteratos A. (2023). Sustainable Crop Protection via Robotics and Artificial Intelligence Solutions. Machines.

[13] Abdulsalam Y.S., Hedabou M. (2022). Security and Privacy in Cloud Computing: Technical Review. Future Internet.

[14] Malgieri G., Pasquale F. (2024). Licensing high-risk artificial intelligence: Toward ex ante justification for a disruptive technology. Comput. Law Secur. Rev..

[15] Truong N., Sun K., Wang S., Guitton F., Guo Y. (2021). Privacy preservation in federated learning: An insightful survey from the GDPR perspective. Comput. Secur..

[16] Agahari W., Ofe H., de Reuver M. (2022). It is not (only) about privacy: How multi-party computation redefines control, trust, and risk in data sharing. Electron. Mark..

[17] Dhanaraju M., Chenniappan P., Ramalingam K., Pazhanivelan S., Kaliaperumal R. (2022). Smart Farming: Internet of Things (IoT)-Based Sustainable Agriculture. Agriculture.

[18] Jakobsen K., Mikalsen M., Lilleng G. (2023). A literature review of smart technology domains with implications for research on smart rural communities. Technol. Soc..

[19] Dargaoui S., Azrour M., Allaoui A., Guezzaz A., Alabdulatif A., Alnajim A. (2024). Internet of Things Authentication Protocols: Comparative Study. Comput. Mater. Contin..

[20] Li M., Zhou S., Shen S., Wang J., Yang Y., Wu Y., Chen F., Lei Y. (2024). Climate-smart irrigation strategy can mitigate agricultural water consumption while ensuring food security under a changing climate. Agric. Water Manag..

[21] Ferrag M.A., Shu L., Djallel H., Choo K.-K.R. (2021). Deep Learning-Based Intrusion Detection for Distributed Denial of Service Attack in Agriculture 4.0. Electronics.

[22] Farooq M.S., Riaz S., Abid A., Umer T., Zikria Y.B. (2020). Role of IoT Technology in Agriculture: A Systematic Literature Review. Electronics.

[23] Mishra A., Alzoubi Y.I., Anwar M.J., Gill A.Q. (2022). Attributes impacting cybersecurity policy development: An evidence from seven nations. Comput. Secur..

[24] Panagopoulos A., Minssen T., Sideri K., Yu H., Compagnucci M.C. (2022). Incentivizing the sharing of healthcare data in the AI Era. Comput. Law Secur. Rev..

[25] Williams P., Dutta I.K., Daoud H., Bayoumi M. (2022). A survey on security in internet of things with a focus on the impact of emerging technologies. Internet Things.

[26] Zahid R., Altaf A., Ahmad T., Iqbal F., Vera Y.A.M., Flores M.A.L., Ashraf I. (2023). Secure Data Management Life Cycle for Government Big-Data Ecosystem: Design and Development Perspective. Systems.

[27] Saxena N., Hayes E., Bertino E., Ojo P., Choo K.-K.R., Burnap P. (2020). Impact and Key Challenges of Insider Threats on Organizations and Critical Businesses. Electronics.

[28] Butpheng C., Yeh K.-H., Xiong H. (2020). Security and Privacy in IoT-Cloud-Based e-Health Systems—A Comprehensive Review. Symmetry.

[29] Kaur J., Fard S.M.H., Amiri-Zarandi M., Dara R. (2022). Protecting Farmers’ Data Privacy and Confidentiality: Recommendations and Considerations. Front. Sustain. Food Syst..

[30] Kaur J., Dara R. (2023). Analysis of Farm Data License Agreements: Do Data Agreements Adequately Reflect on Farm Data Practices and Farmers’ Data Rights?. Agriculture.

[31] Zscheischler J., Brunsch R., Rogga S., Scholz R.W. (2022). Perceived risks and vulnerabilities of employing digitalization and digital data in agriculture—Socially robust orientations from a transdisciplinary process. J. Clean. Prod..

[32] Demestichas K., Peppes N., Alexakis T. (2020). Survey on Security Threats in Agricultural IoT and Smart Farming. Sensors.

[33] Yaacoub J.-P.A., Noura H.N., Salman O., Chehab A. (2023). Ethical hacking for IoT: Security issues, challenges, solutions and recommendations. Internet Things Cyber-Phys. Syst..

[34] Rettore de Araujo Zanella A., da Silva E., Pessoa Albini L.C. (2020). Security challenges to smart agriculture: Current state, key issues, and future directions. Array.

[35] Pandey N.K., Kumar K., Saini G., Mishra A.K. (2023). Security issues and challenges in cloud of things-based applications for industrial automation. Ann. Oper. Res..

[36] Seh A.H., Zarour M., Alenezi M., Sarkar A.K., Agrawal A., Kumar R., Ahmad Khan R. (2020). Healthcare Data Breaches: Insights and Implications. Healthcare.

[37] Javaid M., Haleem A., Singh R.P., Suman R. (2023). Towards insighting cybersecurity for healthcare domains: A comprehensive review of recent practices and trends. Cyber Secur. Appl..

[38] Iqbal M.Z., Campbell A.G. (2022). Potential security and privacy issues in zero UI touchless technology. Int. Cybersecur. Law Rev..

[39] Alawida M., Omolara A.E., Abiodun O.I., Al-Rajab M. (2022). A deeper look into cybersecurity issues in the wake of Covid-19: A survey. J. King Saud. Univ.—Comput. Inf. Sci..

[40] Choudhary K., Gaba G.S., Butun I., Kumar P. (2020). MAKE-IT—A Lightweight Mutual Authentication and Key Exchange Protocol for Industrial Internet of Things. Sensors.

[41] Avanesov T., Chevalier Y., Rusinowitch M., Turuani M. (2017). Satisfiability of general intruder constraints with and without a set constructor. J. Symb. Comput..

[42] Agilandeeswari L., Paliwal S., Chandrakar A., Prabukumar M. (2022). A new lightweight conditional privacy preserving authentication and key—*Agreement* protocol in social internet of things for vehicle to smart grid networks. Multimed. Tools Appl..

[43] Paliwal S. (2019). Hash-Based Conditional Privacy Preserving Authentication and Key Exchange Protocol Suitable for Industrial Internet of Things. IEEE Access.

[44] Thakur G., Kumar P., Chen C.-M., Vasilakos A.V., Anchna, Prajapat S. (2023). A Robust Privacy-Preserving ECC-Based Three-Factor Authentication Scheme for Metaverse Environment. Comput. Commun..

[45] Ashraf Z., Sohail A., Yousaf M. (2023). Robust and lightweight symmetric key exchange algorithm for next-generation IoE. Internet Things.

[46] Manikandan S., Rahaman M., Song Y.-L. (2022). Active Authentication Protocol for IoV Environment with Distributed Servers. Comput. Mater. Contin..

[47] Cherbal S., Benchetioui R. (2023). ScPUAK: Smart card-based secure Protocol for remote User Authentication and Key agreement. Comput. Electr. Eng..

